# Obstetrics and Gynecology

**Published:** 1906-12

**Authors:** E. S. McKee

**Affiliations:** Cincinnati, Ohio


### PROGRESS IN MEDICAL SCIENCE.

### Obstetrics and Gynecology.

By E. S. McKEE, M. D., Cincinnati, Ohio.

### PREGNANCY AFTER CONSERVATIVE OPERATION FOR ACUTE GONORRHEAL SALPINGITIS.

STONE (*Amer. Journ. Obstet.*, May, 1906) writes of a single woman aged 20, admitted into hospital with acute abdominal pains, high pulse and temperature. All the symptoms of peritonitis from appendicitis or salpingitis were present. Her intended husband admitted that he was suffering from gonorrhea, and was responsible for the patient's condition. Abdominal section was performed, extensive peritoneal inflammation discovered; it was traced to the right appendages, which were removed. The uterus and left tube were irrigated with mercuric bichloride, as some pus issued from the ostium of the tube when it was brought out of the wound for inspection. The vermiform appendix was removed, the lower end of the abdominal wound drained, and the patient recovered. Ten months after the operation she was married, and at a date not clearly indicated in the report, but apparently about eight years later, she bore a female child.

### CORNUAL PREGNANCY; RUPTURE; PREGNANCY IN OPPOSITE CORNU AFTER OPERATION.

Doran (*Journ. of Obst. and Gyn. of the Brit. Emp.*, June, 1906) publishes a full report of a case in which, in September, 1904, he removed a fetal sac, consisting of the right uterine cornu, for acute hemorrhage due to its rupture. The patient was 27 years of age, and had borne two children. The placenta and membranes protuded from the rupture in the sac; a fetus 3 in. long was found under the omentum. The sac, with the right Fallopian tube and ovary, was amputated; the left uterine cornu, which had twice been the seat of normal pregnancy, was empty. One year after operation, in September 1905, the patient was spontaneously delivered of a live child at term. Kustner (*Monats. f. Geb. u Gyn.*, May, 1906) relates another case of cornual pregnancy without rupture, where a similar conservative operation was practised. This patient was 32 years of age, and had been pregnant seven times (including four abortions). She believed herself to be six months pregnant, the period having ceased for that space of time. Uterine duplicity with arrested pregnancy or myoma in one horn was diagnosticated and abdominal section performed. The left cornu was removed with the corresponding appendages; it ran into a cervix almost distinct from that connected with the right cornu (uterus pseudodidelphys, rarer than uterus bicornis unicollis). There were no complications during convalescence. The amputated left cornu contained a much flattened fetus of about the sixth month without a trace of amniotic fluid. In neither of the above cases did the cavity of the cornual sac communicate with that of the opposite cornu or with the canal of the cervix.

---

### PERILS OF INTRAUTERINE INSTRUMENTS.

Jakob (*Zent f Gyn. No.* 19, 1906) in a thesis on the dangers of intrauterine instrumental treatment, has collected 141 cases of perforation of the uterus and others of alleged sounding of the Fallopian tube. The perforations were caused by the curet in 73 cases, the sound in 19, the dilator in 16, the ovum forceps in 14, and the nozzle of a syringe in 6. In 64 cases the uterus had been gravid, 30 being abortions, 34 puerperal. In 12 cases instruments were used for metritis independent of gestation, in 7 for malignant growths, in 3 for removal of a polypus, and in 1 the uterus was perforated during the application of the tampon after abortion. In 5 instances of perforation each case was under treatment for retroflexion, in 4 a fibroid uterus was perforated. Rupture of the uterus during pregnancy occurred in 4 cases where there had previously been instrumental perforation. Jakob declares that he has collected 7 authentic cases where the sound entered the tube, 23 out of the 141 cases of perforation ended fatally, mostly through septic peritonitis, whilst in 77 no grave symptoms were noted. When danger threatens the patient after an intrauterine wound, abdominal section, with or without extirpation of the damaged uterus, is indicated. In some cases the iodoform gauze tampon, or even opiates and applications of ice, have proved sufficient. In 2 cases where spontaneous healing occurred abdominal section was necessary afterward on account of extensive adhesions.

---

LABOR OBSTRUCTED BY UNRUPTURED HYMEN.

DR EDWIN CHILL (Ealing, W.) writes: The following case, which came under my care some time ago in North London, (*British Med. Journ.,* July 28, 1906) is worth recording. With an experience of over 3,000 labors during the last twenty-three years, this is the only example of the kind I have had, though similar instances are mentioned in obstetrical works. The patient was a young woman who had been married about a year. When summoned to attend, labor had progressed for many hours, for the fetal head was well down on the perineum. The hymen was firm, rigid, and unyielding, and would not admit even the little finger. As this condition was obstructing labor, I made several incisions into the hard, fibrous material, after which delivery proceeded naturally. The perineum was slightly lacerated, but this was purposely left unsutured. The presentation was normal; no complications followed, and the mother subsequently did well.

---

EMBRYOTOMY ON THE LIVING INFANT

PIERRE BUDIN (*L'Obstetrique,* May, 1906) describes a case in which to save the life of the mother embryotomy on her living child was carried out. In this case the woman possessed a Nægle pelvis and efforts to deliver by forceps were fruitless. The woman made a good recovery from the operation. He gives the opinions of many of the leading obstetricians as to whether embryotomy on a living child is justifiable or not. The majority of these agree that in certain cases the proceeding is quite justifiable and is especially indicated in those cases in which Cesarean section or symphysiotomy, even if successfully carried out, would not suffice to save a child the life of which had already been considerably imperiled by the prolonged labor.

---

WILLIAM J. ROBINSON (*Critic and Guide,* March, 1906) in a paper on "Proprietary Remedies from the Physician's Standpoint": Urotropin was a distinctly new drug when introduced by its manufacturers, and they and Nicolaier have rendered a real service to humanity. Why should they not derive pecuniary benefit? I am not at all sure that every nondescript brand is as pure as urotropin, and the patient has to pay the same price.

